# Relations among appetitive aggression, post-traumatic stress and motives for demobilization: a study in former Colombian combatants

**DOI:** 10.1186/1752-1505-7-9

**Published:** 2013-04-10

**Authors:** Roland Weierstall, Claudia Patricia Bueno Castellanos, Frank Neuner, Thomas Elbert

**Affiliations:** 1 Clinical Psychology and Neuropsychology, University of Konstanz, Konstanz, Germany; 2 Clinical Psychology and Psychotherapy, University of Bielefeld, Bielefeld, Germany

**Keywords:** Appetitive aggression, PTSD, Colombian former combatants, Resilience

## Abstract

**Background:**

Former combatants have frequently reported that aggressive behaviour can be appetitive and appealing. This appetitive aggression (AA) may be adaptive for survival in a violent environment, as it is associated with a reduced risk of combat-related psychological traumatization. At the same time, AA might impair motivation for re-integration to civil life after ending active duty. Whereas in Colombia those combatants who volunteered for demobilization were mostly tired of fighting, those who demobilized collectively did so mainly by force of the government. We predicted those who were demobilized collectively would still be attracted to violence, and benefit from the resilience against trauma-related mental suffering, moderated by appetitive aggression, as they would have continued fighting had they not been forced to stop.

**Method:**

A sample of 252 former Colombian former combatants from paramilitary and guerrilla forces was investigated. Appetitive aggression was assessed using the Appetitive Aggression Scale (AAS) and post-traumatic stress disorder (PTSD) symptoms with the PTSD Symptom Scale-Interview (PSS-I). We distinguished between individual and group demobilization and assessed reasons for disarmament.

**Results:**

Most of the guerrilla troops who demobilized individually and were tired of fighting reported both an attraction to violence as well as increased trauma symptoms, owing to their former engagement in violent behaviour. In contrast, among those who were demobilized collectively, appetitive aggression was associated with a reduced risk of PTSD. However, this effect was not present in those combatants in the upper quartile of PTSD symptom severity.

**Conclusion:**

The influence of combat experience on traumatization, as well as the motivation for demobilization, differs remarkably between those combatants who demobilized individually and those who were members of a group that was forced to demobilize. This has important implications for the implementation of re-integration programmes and therapeutic interventions.

## Background

Aggressive behaviour is a complex phenomenon, and many theories have been put forth attempting to describe its nature and explain its origins. The most common and well-established approach is to define aggressive behaviour based upon its underlying motivation, distinguishing between *reactive aggression* and *instrumental aggression*. While reactive aggression has been defined as any automatic and emotional aggressive behaviour that occurs as a response to a perceived threat or provocation [1,2], instrumental aggression relates to aggressive behaviour with the purpose of achieving certain goals or gaining social status [3,4]. Although this distinction has been repeatedly criticized [5], meta-analyses have confirmed that human aggressive behaviour has two different facets [6], and that a distinction must be made. Undoubtedly, there is a reactive form with the purpose of repelling a particular threat. This is associated with a high state of arousal and a negative emotional state. It is the struggle to reduce this negative arousal that motivates and drives this aggression. This form of aggression has been examined extensively in numerous laboratory and field studies. In contrast to this, another form of aggression, termed ‘appetitive aggression’, has been put forth in recent years, based on anthropological observations of cruel human behaviour [7,8]. This is not driven by self-defence or secondary rewards, such as resources or status, but is motivated by the primary intrinsic enjoyment of the aggressive act itself. Appetitive aggression increases positive arousal and seems rewarded by the exposure to cues of violence, like the suffering of the victim or the preparation for hunting down humans [9]. Our research group has examined appetitive aggression in many different studies from a variety of different populations, including the Democratic Republic of Congo (DRC), Rwanda, Uganda, and also with German World War II veterans, encompassing more than 2000 participants. We found that appetitive aggression, conceived as the perpetration of violence and/or the infliction of harm to a victim for the purpose of experiencing violence-related heroism and enjoyment, is indeed very common amongst former combatants. In these studies, we administered the Appetitive Aggression Scale (AAS, [9]), which focuses particularly upon a sense of positive arousal (e.g. ‘Is it exciting for you if you make an opponent really suffer?’, ‘Is defeating the opponent more fun for you, when you see them bleed?’, ‘Once you were used to being cruel, did you want to be crueller and crueller?’). Appetitive Aggression was so high in some of the participants that they even reported a craving to behave cruelly during their time in combat. Thus, besides the secondary rewards that can be gained as a consequence of aggressive behaviour in general, the perpetration of violence itself can be self-rewarding and facilitate the outbreak of cruelty.

Moreover, besides the aforementioned appetitive reward, the disposition to aggressive behaviour has an additional beneficial characteristic in regard to mental health and is related to the processing of cruelty: An actual or threatened death, serious injury, or threat to the physical integrity of self or others is potentially traumatic. The killing of a victim might therefore fulfil the diagnostic criteria for a traumatic event according to DSM IV. As shown by a number of studies with victims of violence, the greater the exposure to traumatic event types, the greater the risk for the development of trauma-spectrum disorders, including posttraumatic stress disorder (PTSD; [10]). This is not a simple dose–response effect, as the variety of traumatic stressors also counts, and thus the effect has been termed the ‘building-block-effect’. While it leads to widespread mental suffering amongst victims, this does not appear to be the case for perpetrators [7]. Consequently, there has to be a protective mechanism that prevents the perpetrator from becoming mentally ill and dysfunctional in response to their violent behaviour. Our previous investigations suggest that it is appetitive aggression that reduces vulnerability for traumatic stress [11,12]. Perpetrators seem to be able to tolerate a greater exposure to violence and traumatic stressors, and will only suffer from PTSD after having been exposed to an extremely high amount of trauma [13].

After one of the longest internal armed conflicts in the world, among guerrilla groups, paramilitary organizations and the Colombian army, Colombia is trying, through systematic demobilization of the illegal groups, to build peace. The two largest guerrilla groups in Latin-America, Fuerzas Armadas Revolucionarias de Colombia (FARC) and Ejercito de Liberacion Nacional (ELN), as well as the paramilitaries and anti-guerrilla group (AUC) have been involved in serious atrocities like killings, massacres, kidnappings or the use of landmines and booby traps. Amnesty International estimates that, in the past 20 years, more than 70,000 people have been killed, while thousands have been kidnapped, tortured or forcibly abducted to serve in one of the armed forces [14]. In 2002, the government started negotiations with the paramilitary forces and guerilla groups, to promote the individual demobilization of combatants from all groups, and the collective demobilization for the paramilitary forces Laplante & Theidon, (2006, [15]) focusing on individual demobilization. One idea of these Disarmament, Demobilization and Reintegration (DDR) programmes is that the government has to create an attractive environment for former combatants, by fostering physical security and economic support to prevent future engagement in violent behaviour [16,17]. As aggressive acts performed in mass violence are mostly found to be reward-driven [18], material rewards should reduce instrumentally used aggression. However, Theidon [14], who interviewed more than 100 former Colombian combatants, points out that, irrespective of the harsh reality, ‘militarized masculinity’ is one of the desirable rewards associated with a warrior’s life. Consequently, non-material rewards associated with combat, cannot be outweighed by money and might still arouse aggressive behaviour after demobilization. One further challenging issue is that there are two forms of demobilization in the Colombian demobilization process: On an individual level (mainly guerrilla fighters), as well as collective demobilization (mainly paramilitary forces). Correspondingly, there are former combatants who demobilized voluntarily because they were tired of fighting [19], while others just demobilized because of an agreement between the government and the forces’ leaders, without necessarily wanting to give up fighting. Combatants from both groups – paramilitary and guerrilla forces – tried to leave the force when they were tired of fighting, often because their lives were under threat. However, collective demobilization was primarily a consequence of negotiations between the government and the paramilitary.

With this study, we wanted to investigate whether appetitive aggression is still present among Colombian ex-combatants. We hypothesized that appetitive aggression would be prominent in those who were demobilized by force, as the attraction to violence would have contributed to their participation in armed groups. Moreover, because these combatants were not driven by intrinsic motivation to lay down their weapons, we tested the hypothesis that they would still be attracted to violence and benefit from the protective effects of appetitive aggression on traumatic stress. In contrast, we hypothesized that this effect would be weak or absent in those who demobilized individually and voluntarily. We expected them to no longer experience a protective effect of appetitive aggression. We assessed appetitive aggression in two groups of former combatants, one that was demobilized collectively by force and one in which the ex-combatants joined the demobilisation program on their own initiative. To test the first hypothesis, we compared appetitive aggression between these two groups. To investigate the relation between appetitive aggression and trauma load on the severity of PTSD symptoms, we calculated a moderation analysis.

## Methods

### Setting

Data were collected in the north-east of Colombia between June 2009 and May 2010, as part of a larger study. Participants were recruited from a reception camp for former combatants who had been demilitarized. The government, the leading armed forces, and guerilla and paramilitary forces have been in the process of peace negotiations for several years. The demilitarization camp has been designed as a first drop-in centre for former combatants on their way to re-socialization. Participants were recruited through the state programme for social and economic reintegration of illegal armed insurgent groups (High Council for Reintegration; ‘ACR’ in Spanish). The ACR was created in 2006, by the Colombian Government, under the direction of the Ministry of Justice. The ACR prepares the demobilized for reintegration into society through psychosocial, academic training and access to the national health system. ACR provides monthly financial assistance to active participants in the demobilization programme.

### Participants

The interviews took place in Bucaramanga, the capital city of the department of Santander, Colombia, which has the fifth biggest re-integration centre in the whole of Colombia. We chose all 252 combatants from a population of about 600 combatants in the premises of the ACR, who had to attend psychosocial meetings during the time of data collection. These meetings are obligatory to all combatants in the process of demobilization. After demobilizing, the former combatants are transferred to one of the re-integration centres and distributed across the whole country. Santander has no specific re-integration focus so that all former combatants who demobilize have about equal probability of being transferred to Santander. Therefore, there is no selection bias and the participants are maximally representative for the population of former Colombian combatants. 83% (n = 209) of the interviewed demobilized were male, 17% (n = 43) female. The average age of men was 31.24 (SD = 7.8) and for women 28.65 (SD = 7.8) years. 23% (n = 58) belonged to FARC, 18.3% (n = 46) to ELN, and 55.2% to the AUC paramilitary groups. The objectives of the investigation, strict confidentiality, as well as the right to refuse to participate at any time in the interview, without any consequences, were explained in detail to the participants. All participants gave written informed consent, indicating their informed willingness to participate. All completed the interview. The Ethical Review Board of the University of Konstanz, and the ACR authorities, approved the study protocol. The interviews were carried out with support of the ACR, whereby strict confidentiality was ascertained. Participants did not receive any financial compensation or other direct benefit.

As we were interested in group differences between participants who had either been demobilized in groups or on an individual basis, we grouped all participants by their means of demobilization into *collective demobilization* or *individual demobilization*. A Chi-Square test for a fourfold table, with the two factors *demobilization* (collective demobilization vs. individual demobilization) and *armed group* (paramilitary vs. guerrilla) was calculated. Most of the participants from the paramilitary forces had been demobilized in groups (collective demobilization: n = 90), whereas only about one third demobilized of their own accord (individual demobilization: n = 49). The opposite was true for the guerrilla forces: Only 11 of them reported that they had been demobilized collectively. The majority (n = 93) demobilized individually. This difference in demobilization between groups was statistically significant (*X*^2^_1_ = 71.88, *p* < .001) indicating that this factor is a crucial difference between the two groups.

### Local team

A team of Columbian clinical psychologists carried out the interviews. The team leader had been trained at the University of Konstanz’s Centre For Psychotraumatology, and another six experienced clinical psychologists received two weeks’ training in the concepts of PTSD, aggression, the principles of quantitative data collection in semi-structured clinical interviews, as well as interviewing techniques. All research experts and interviewers were supervised at least once a week throughout the study. The supervision included help with distressing emotional experiences during the data collection, as well as the discussion of the quality of their work, to guarantee maximum validity and interrater-reliability.

### Instruments

#### Severity of trauma exposure

We administered a checklist of 35 different traumatic event types for the assessment of the severity of trauma exposure. This checklist [20] has already been administered in several other samples of combatants (e.g. [12]). It included five items for domestic violence. Events that either could have been self-experienced or witnessed, were scored as *present* or *not present* in the participants’ lives, and summed up to a *traumatic event types* score. Participants reported that they had experienced 1 to 22 different traumatic event types. The average number of reported traumatic event types was nearly identical for the participants in the collective demobilization (*M* = 9.9, *SD* = 4.7) and for those in the individual demobilization group (*M* = 9.9, *SD* = 4.4; independent sample *t-*test: *t*_250_ = .08, *p* = .94, *d* = .00).

#### PTSD symptom severity

We assessed the DSM-IV diagnosis and *PTSD symptom severity* with the PSS-I (PTSD Symptom Scale Interview; [21]). The PSS-I is a semi-structured interview designed to assess current symptoms of PTSD. It consists of 17 questions that correspond to the DSM IV diagnostic criteria, including intrusions, avoidance and hyperarousal, and refers to a two-week period. The valid assessment of PTSD symptoms in non-Western populations, using structured interviews, has been demonstrated in several publications, and has demonstrated satisfying psychometric properties [10,22,23]. The PSS-I score, as a measure of PTSD symptom severity, was calculated as the sum over the item-scores on the scale, and thus had a range from 0 to 51 points. There was no significant difference in the mean scores between the two groups (group-demobilization: *M* = 15.3, *SD* = 9.7; individual demobilization: *M* = 15.7, *SD* = 9.7; independent sample *t-*test: *t*_250_ = .08, *p* = .94, *d* = .04).

#### Attraction to violence

For the assessment of a person’s propensity to violence, we administered the Appetitive Aggression Scale (AAS; [9]). The AAS assesses enjoyment related to violence-cues in combatants. It consists of 15 statements that have to be rated as either true or not true, on a 4-point Likert-scale ranging from 0 (‘I totally disagree’) to 4 (‘I totally agree). The scale has been administered to over 1600 participants in different war-affected populations and has proven its good psychometric properties [9]. We used the AAS score, which ranged from 0 to 59 points, to measure *attraction to violence*. The score lay in the range of other combatant populations from Uganda or The Democratic Republic of Congo [9].

#### Reason for demobilization

We hypothesized that the influence of appetitive aggression on trauma symptoms might be different between participants from individual and collective demobilization. To assess the *reason for demobilization,* participants were asked to choose one of four different categories, based on the literature on demobilization in Colombia, that best characterized their motivation for demobilization. The two most prevalent reasons from the literature were *tired of fighting* (owing to mental health problems or bad living conditions) and *political force* (i.e. an order from the commander)*.* However, we also asked about *family problems* that forced the combatant to leave the forces as well, such as *life threat*, i.e. when the combatants feared for their lives if they stayed with the forces.

#### Procedure

All interviews were conducted in Spanish. For this purpose we used back-and-forth translations from English to Spanish, including blind back-translations and subsequent corrections by different expert groups. For all items of the survey, the interviewers made further inquiries, or gave examples of symptoms, and probed the answers to ensure a correct understanding of the concepts and maximize data validity, especially with respect to the clinical significance of PTSD symptoms.

#### Data analysis

In line with our research question, we hypothesized that the relation between the traumatic event load and the PTSD symptom severity (building-block effect) is moderated by appetitive aggression scores. To test this hypothesis, we regressed the PTSD symptom severity on the severity of trauma exposure, the attraction to violence, a dichotomous dummy variable for the manner of demobilization (‘0’ = individual demobilization; ‘1’ = collective demobilization), as well as on all possible one- and two-way interactions and squared terms. All variables were mean-centered before interaction-terms were calculated to mitigate multi-collinearity. The Akaike Information Criterion (AIC; [24]) was used as a measure for model fit. For a clearer representation of moderation and mediation effects within the two groups, we performed further separate post-hoc analyses. Statistical modelling was conducted using linear regression analyses with SPSS 19 for Mac.

## Results

### Difference in appetitive aggression between groups

To test for differences in the attraction to violence between participants that were demobilized individually and those were demobilized collectively, we calculated an independent-sample *t*-test for group differences. As in the two other measures, the trauma symptom severity and the severity of trauma exposure, the score did not differ significantly between groups (collective demobilization: *M* = 15.1, *SD* = 12.8, individual demobilization: *M* = 14.7, *SD* = 11.3; independent sample *t-*test: *t*_250_ = .24, *p* = .81, *d* = .09). Thus, against our prediction, participants from both groups seem to have a comparable level of appetitive aggression.

### The protective effect of appetitive aggression and its relation to the manner of demobilization and PTSD symptom severity

In the first linear regression analyses of PTSD symptom severity on the attraction to violence, the severity of trauma exposure, the group dummy variable and all possible interactions, the final model selection, according to AIC, revealed that the best fitting model only included the two variables, attraction to violence and exposure to traumatic stressors. As both had significant positive beta values (attraction to violence: *β* = .25, *p* < .001; severity of trauma exposure: *β* = .35, *p* < .001; *F*_2, 245_ = 45.67, *p* < .001, *R*^*2*^_adj_ = .27, *f*^*2*^ *=* .37, (1-*β*) = 1.00), there seemed to be no protective effect of an appetitive perception of aggression in this sample, compared to other combatant populations.

However, as we discovered in our previous studies, appetitive aggression only provides resilience against traumatic stress as long as the PTSD symptom severity has not exceeded a certain threshold, we performed the same analyses after excluding those 65 participants in the upper quartile of PTSD symptom severity (PSS-I score > 21). The selected regression model (*F*_6,181_ = 6.14, *p* < .001, *R*^*2*^_adj_ = .17, *f*^*2*^ *=* .21, (1-*β*) = 1.00) included all three predictor variables as well as the three one-way interactions (Table 1). The two-way interaction did not reach statistical significance.

**Table 1:** Linear regression analysis predicting trauma severity from the severity of trauma exposure, appetitive aggression, the participant’s group and all possible two-way interactions (n = 184)

	**PDS score**	
	***β***	***p***
Severity of trauma exposure	.49	.049
Appetitive aggression	.10	< .001
Group (way of demobilization)	1.43	n.s.
Interaction: severity of trauma exposure * attraction to violence	- .01	n.s. (.09)
Interaction: attraction to violence * group	- .15	.031
Interaction: severity of trauma exposure * group	.25	n.s.

**Note.** Uncorrected standardized regression coefficients are displayed. The group dummy variable reflects the type of demobilization (‘0’ = individual demobilization; ‘1’ = collective demobilization).

As indicated by the negative interaction between the attraction to violence and the group dummy variable, there is a protective effect of appetitive aggression on the PTSD symptom severity in participants who were demobilized in groups, compared to those who demobilized themselves on an individual level. Moreover, even if it is not statistically significant on a 5% level, there is a trend for the negative interaction between the severity of trauma exposure and the attraction to violence. However, considering that the beta-value of -.01 for the latter interaction is small, these two interaction terms suggest that the protective effect found in other ex-combatant groups is masked in the present data set by those who had demobilized individually. The non-significant interaction between the severity of trauma exposure and the group dummy indicates that the building-block effect may not differ between groups. Besides the interactions, there was a major effect for severity of trauma exposure displaying the building-block effect, and a main effect for attraction to violence. We investigated the latter in more detail:

### The protective effect of appetitive aggression in the group-demobilization group

**Figure 1:**
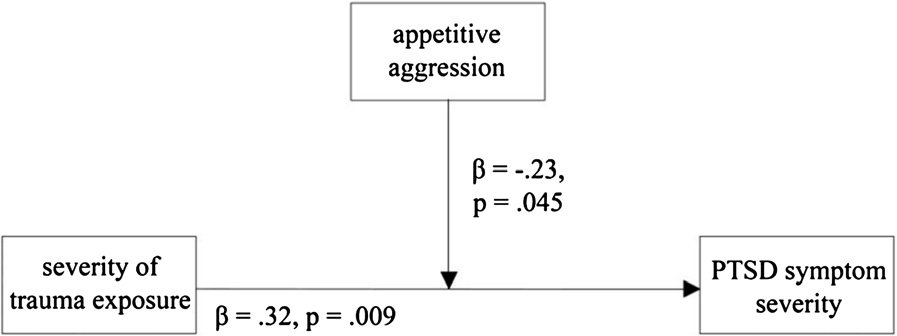
Moderation analyses for the relation among the traumatic event types score, the attraction to violence and the PSD symptom severity in participants that demobilized in groups (n = 74).

To investigate the inverse relation between attraction to violence and PTSD symptom severity further, we performed separate regression analyses for both groups. Taking into account the previously reported building-block effect (Neuner et al., 2004), which implies trauma-related symptoms in all survivors when exposure to traumatic stress is massive, participants from the upper quartile of PTSD symptom severity were excluded from the analyses. In the collective demobilization group, we regressed the PTSD symptom severity on the attraction to violence and the traumatic event types score, as well as on the interaction between both predictor variables. The regression model (*F*_3,71_ = 4.99, *p* = .003, *f*^*2*^ = .20, (1-β) = .90) revealed that the building-block effect, which is indicated by the increasing likelihood of PTSD with increasing exposure to traumatic event types, is moderated by the attraction to violence (see Figure 1), whereas there is no direct effect of attraction to violence on PTSD symptoms (*β* = .17, *p* = .152).

There was no problem with collinearity in the proposed model (maximum Variance Infamation Factor (VIF) = 1.18). The residuals did not differ significantly from normal distribution (Kolmogorov–Smirnov *Z* = .48, *p* = .973). Moreover, there was no severe influence of outliers in the proposed models (maximum Cook’s d of .371). However, if the 30 participants that fall into the upper quartile of the PTSD symptom severity were added to the calculation, the protective Effect vanished (Interaction severity of trauma exposure * attraction to violence: *β* = .02, *p* = .957). The only significant predictor was the severity of trauma exposure (*β* = .48, *p* = .044), while the attraction to violence still had no significant impact on PTSD symptom severity (*β* = .15, *p* = .489; *F*_*3,101*_ = 16.88, *p* < .001, *f*^*2*^ = .50, (1-β) = 1.00).

### The relation between traumatic stress and attraction to violence in the individual demobilization group

**Figure 2:**
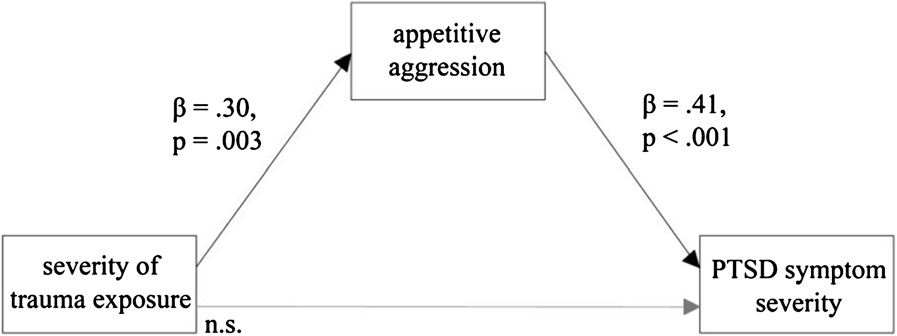
Mediation analyses for the relation among the traumatic event types score, the attraction to violence and the PSD symptom severity in participants that demobilized individually (n = 106).

To investigate the relationship between exposure to traumatic stressors and attraction to violence, in the group of participants that demobilized themselves individually, we performed the same analyses as for the group demobilization sample. As the interaction term between attraction to violence and traumatic event types score did not reach statistical significance (*β* = −.05, *p* = .811), it was excluded from the regression model in a first step. Surprisingly, the regression model (*F*_2,104_ = 10.90, *p* < .001, *f*^*2*^ = .21, (1-β) = .90) revealed that the traumatic event types score was not a significant predictor of PTSD symptom severity (*β* = .10, *p* = .240). Instead, the building-block effect was completely mediated by the attraction to violence (Figure 2), which was the only significant predictor. This relation changed when the 35 participants from the upper quartile of PTSD symptom severity were added to the calculation. In a regression analysis where we regressed the symptom severity on the attraction to violence and the severity of trauma exposure, both predictors turned out to be statistically significant (attraction to violence: *β* = .30, *p* < .001; severity of trauma exposure: *β* = .30, *p* < .001; *F*_2,140_ = 22.01, *p* < .001, *f*^*2*^ = .20, (1-β) = .90).

### Group differences in the reasons for demobilization

As reported earlier in this manuscript, the two groups, paramilitary and guerrilla forces, differed significantly in their way of demobilization. To validate our group assignment and link the way of demobilization to the underlying motivation, we compared the distribution in the answers for the *reasons for demobilization* between groups. As can be seen in Table 2, from two-thirds of all answers, most former combatants that demobilized themselves individually were tired of fighting. The opposite was true for participants that experienced collective demobilization. They most often reported that the only reason for dispensing with their weapons was an order from their commanders (94%). The difference in the distribution of the answers between groups was statistically significant (*χ*^*2*^_*3*_ = 196.62, *p* < .001).

**Table 2:** Differences in the reasons for demobilization between groups (n = 245)

	**Number of responses (percent)**	
	**Individual demobilization**	**Collective demobilization**
Tired of fighting	91 (63%)	6 (94%)
Political force	8 (6%)	100 (6%)
Family problems	30 (21%)	0
Life threat	15 (10%)	0

#### Gender differences

From our 252 participants, at least 17% were female. To test for gender differences, the factor *gender* was added as a 0–1 coded dummy variable to the regression analysis in the two subgroups, as well as all possible interaction terms with the significant predictor variables. However, the factor gender did neither reach statistical significance (*p* > .05 for all *β*) in any of the analyses, nor did it lead to significant *R*^2^-changes. Thus, the female former combatants in this sample did not differ in both groups from their male comrades in the relation between an appetitive processing of aggression and the severity of PTSD symptoms.

## Discussion

The aim of the present study was to investigate differences in the relation of appetitive aggression and traumatic stress between former Colombian combatants who either demobilized collectively or individually. We obtained four major results: (1) Appetitive Aggression is common amongst Colombian former combatants, regardless of whether they demobilized individually or collectively. We could not detect a significant difference between groups. (2) Members of both groups reported a similar range of PTSD symptoms. (3) In line with previous results, the protective effect of an appetitive perception of violence on PTSD symptoms could be documented in those who were demobilized collectively, but seemed to be absent in those who demobilized individually. (4) We also note that the resilience wanes when the severity of exposure to traumatic stress exceeds a certain threshold. In fact, those who suffered from very serious trauma symptoms reported that they experienced cruelty to others as self-rewarding.

### Addressing appetitive aggression

The wide extent of appetitive aggression across the population that we obtained in this study, as well as in earlier studies, contradicts the hypothesis that human predation is limited to a pathological subgroup, such as psychopaths [25]. Indeed, it seems to be a common facet of human behaviour, which surfaces in the context of war [9]. Cruelty becomes self-rewarding and further increases a combatant’s desire to hunt and kill. Some of the respondents have reported an addiction-like craving to behave cruelly. Consequently, those who are attracted to aggression, and who experience fewer trauma symptoms through a better adaptation to a cruel environment, may have fewer incentives to quit their membership of an armed group. The results from this study suggest that collective demobilization by force does not seem to alter this attraction to fighting. The fact that two-thirds of paramilitaries in this sample demobilized in groups and still seem to benefit from an intrinsic motivation to show violent behaviour corresponds with recent findings that former paramilitaries, in particular, reform in illegal groups and relapse into crime [26]. These groups engage in criminal activities and even continue the tradition of paramilitarism. Only those who were traumatized or suffered from their experiences in combat had the motivation to seek out a demobilization programme on their own. For DDR programmes, we therefore suggest that, besides addressing trauma-related mental illness, it may be useful to also take into account appetitive aggression, as both trauma symptoms and a low threshold for aggression are common amongst former combatants, affecting the potential for re-socialization. In particular, when demobilized collectively, owing to an agreement between the group leaders and the government, former combatants might be screened for high scores on appetitive aggression. A protective context that offers material rewards can be a potentially helpful prerequisite (although not yet proven; see [27]). However, the perpetration of violence with its self-rewarding properties facilitates a cycle of violence, irrespective of material rewards [28] and thus requires specific psychotherapeutic intervention. We have, therefore, developed an intervention programme for the treatment of appetitive aggression [29], which is showing promising initial results.

### Appetitive aggression, traumatic stress and its relation to motivation for demobilization

The relation between the exposure to traumatic stressors during combat and the development of PTSD symptoms has also been reported extensively for those populations who perpetrated violent acts. Many of the research findings stem from US veterans and demonstrate that PTSD symptoms increase in proportion to proximity to the battlefield [30,31]. Contrary to the hypothesis that every kind of perpetrated violence increases the risk of traumatization [32], and in line with the vast majority of research on human intra-species killing, which emphasizes the benefits of hunting down the out-group, evolution may have led to a protective mechanism that prevents perpetrators from being traumatized by their committed atrocities [7]. There is evidence that those perpetrators who were more attracted to violence cues also experience fewer PTSD symptoms [11]. In line with a recent study by Hecker and colleagues [13], the protective effect in this study was only present when the PTSD symptom severity did not exceed a certain threshold. Consequently, appetitive aggression buffers the risk of traumatisation but cannot provide ultimate resilience. Numerous studies in violence-affected regions have studied the consequences of cumulative exposure to traumatic stress, and revealed a strong monotonic relationship between the number of different traumatic event types experienced and the risk of the development of post-traumatic stress disorder [33-35]. Contrary to this traumatic processing, violence cues can be processed differently and appetitively during the perpetration of atrocities [36].

However, with ever increasing exposure to traumatic stress, the protective effect seems to fade. The participants from the group of collective demobilization, in the upper quartile of PTSD symptom severity, no longer displayed an inverse relation between appetitive aggression and trauma symptoms. But what is the underlying mechanism? Research with victim’s of violence revealed a strong relationship between the number of different traumatic event types experienced and the risk of developing post-traumatic stress disorder (PTSD; [28,33]): Events that are processed traumatically form a neural network that contains a person’s cognitions and psychophysiological as well as behavioural information. These elements of an event are linked to the affective meaning of the traumatic event but detached from contextual information. Every new traumatic event type leads to an extension of the fear network [37,38] and increases the risk to be activated and produce PTSD typical symptoms [20]. In severe acts of violence, there is an appetitive perception of violence amongst combatants on the one hand, but every combat bares the risk of being killed or injured on the other hand and confronts combatants with witnessing members of their own group to be killed. Thus, even appetitive events contain a number of potentially fear-eliciting stimuli. We propose that the appetitive stimuli form an associative network as well, referred to as ‘hunting network’ (positive valence), which competes with the fear/trauma network (negative valence) for the mnemonic representation of stimuli that could be integrated into both networks (see [7] for further details). However, it seems that at a certain level of PTSD symptom severity, the underlying fear-network outdoes the appetitive hunting network, the protective effect of appetitive aggression vanishes and cues that formerly belonged to the appetitive hunting network now connect with the aversive fear-network: Physiological responses like heart racing, sensory cues like seeing the blood of the victim and hearing their distressed vocalizations, together with the intense emotions and cognitions related to combat, lose their appetitive connotations. Instead, they connect with the fear responses, associated with traumatic events stored in the brain in the associative fear-network (negative valence). Perpetration of violence can therefore also lose its protective effect. The protective effect we obtained in the sample of those who were demobilized collectively was absent in those who were demobilized individually and were mostly tired of fighting. In the latter group, the relationship between appetitive aggression and trauma symptom severity was even reversed; i.e. those who reported that they experienced violence to be appetitive also showed more severe trauma symptoms. Moreover, most of them reported being tired of fighting, or wanting to go back to their families, and might have had an intrinsic motivation to leave the forces and to avoid the combat-associated stress. In contrast, many of those who were demobilized by force still remained in the ‘perpetrator mode’, benefiting from a self-rewarding appetitive perception of violence that also prevents trauma-associated fear-responses. We suggest that the motivation for demobilization and its associated relations to markers of mental health might therefore be a strong indicator for appraising the psychological needs to be addressed in a DDR program. DDR programmes are designed to facilitate reintegration strategies for return into civil society, and usually do so by addressing the combatant’s needs and aspirations, including medical treatment [39,40]. The psychological condition of the beneficiaries may be the essential predictor of successful long-term reintegration and might add significantly to the effect of DDR programs that mainly focus on socioeconomic variables. The re-engagement of ex-combatants in organized violence is a common phenomenon in post-conflict regions that impairs re-integration not only in Colombia but also in other countries like Afghanistan, Ivory Coast or Liberia [41-44]. We propose that traumatic stress symptoms and appetitive aggression should be examined further in the course of the demobilization process to evaluate appetitive aggression as one significant mediator for future violent behaviour besides material rewards. This psychological issue cannot be neglected in re-integration programs, especially in light of the evidence that even in 2010 a significant proportion out of those who attended the demobilization program has turned to crime again [27,45].

One last important finding is that we did not find any gender differences between female and male former combatants. This might be surprising, as in civil populations dramatic gender differences have been reported in aggression literature [5,46]. However, the selection of women who join armed groups may be special and there is no empirical data on gender differences in aggression in combatant populations available. Our results provide evidence that a selective proportion has either an intrinsic or acquired potential for behaving violently.

### Limitations

Former Colombian combatants experience continuous stress, including potential threat to their lives. The government does not grant amnesty for committed violent crimes [47]. Moreover, some participants in this study were afraid of persecution by their former comrades, as those who left the armed groups were threatened with death. These conditions might have added to the group differences. One further issue is the problem of non-random attrition. Even though the levels of appetitive aggression in this sample are comparable to other populations of former combatants (see [36]), combatants with a high propensity towards violence could have already returned to arms, not engaging in the demobilization program any more. In their latest fact sheet, published in 2009, the office of the Presidential High Counselor for Reintegration revealed the statistic that out of 51.852 persons who expressed their willingness to demobilize between August 2002 and September 2009, only 60% participated in the demobilization program. This could have led to an underestimation of the extent of appetitive aggression in the sample of paramilitary or guerrilla forces, as not all cases can be recorded. However, these cases would have rather increased the effect size of the protective effect so that even a potential selection bias would not contradict the main findings. Moreover, as we did not collect longitudinal data, we cannot exclude the possibility that the groups might already have been different before the war, especially as we find differences between guerrilla and paramilitary groups that stem from different populations within Colombia. Longitudinal data that focuses on differential changes between appetitive aggression and PTSD would help to better understand the dynamic processes that might underlie the processing of violence and mental health symptoms.

## Conclusion

Aggressive behaviour, especially appetitive aggression, as well as traumatization, both profoundly affect former combatants. In combatants that have become tired of fighting and lost their motivation to engage in future violent behaviour, any residual attraction to violence may actually promote PTSD symptoms. In contrast, in those who were demobilized by force, appetitive aggression serves as a resilience factor against the development of PTSD. However, at a certain threshold of exposure to traumatic stressors, this beneficial effect can no longer be observed. This relation between the motivation for violent behaviour and the impact of traumatic stressors needs be considered in DDR programs.

## Competing interests

The authors declare that they have no competing interests.

## Authors’ contributions

RW: Data analysis and manuscript preparation. CB: supervision and training of local psychologists, data collection, analysis and manuscript preparation. FN: data analysis and manuscript preparation, principal investigator. TE: study design, manuscript preparation and supervisor.

## References

[B1] Berkowitz L Aggression: Its causes, consequences, and control 1993 New York: McGraw-Hill

[B2] Schmitt WA Newman JP Passive avoidance in psychopathic offenders: a replication and extension J Abnorm Psychol 1998 107 527 532 9715587 10.1037//0021-843x.107.3.527

[B3] Kruger DJ Nesse RM Sexual selection and the male: female mortality ratio Evol Psychol 2004 2 66 85

[B4] Shackelford TK An evolutionary psychological perspective on cultures of honor Evol Psychol 2005 3 381 391

[B5] Anderson CA Bushman BJ Human aggression Annu Rev Psychol 2002 53 27 51 10.1146/annurev.psych.53.100901.135231 11752478

[B6] Vitielo B Stoff DM Subtypes of aggression and their relevance to child psychiatry J Am Acad Child Adolesc Psychiatry 1997 36 307 315 10.1097/00004583-199703000-00008 9055510

[B7] Elbert T Weierstall R Schauer M Fascination Violence: on mind and brain of man hunters Eur Arch Psychiatry Clin Neurosci 2010 260 2 100 105 10.1007/s00406-010-0144-8 20938671

[B8] Nell V Cruelty’s rewards: The gratifications of perpetrators and spectators Behav Brain Sci 2006 29 211 257 17214016 10.1017/s0140525x06009058

[B9] Weierstall R Elbert T The Appetitive Aggression Scale Eur J Psychotraumatology 2011 2 8430 10.3402/ejpt.v2i0.8430 PMC3402137 22893817

[B10] Neuner F Schauer M Karunakara U Klaschik C Robert C Elbert T Psychological trauma and evidence for enhanced vulnerability for posttraumatic stress disorder through previous trauma among West Nile refugees BMC Psychiatry 2004 4 34 10.1186/1471-244X-4-34 PMC529265 15504233

[B11] Weierstall R Schaal S Schalinski I Dusingizemungu JP Elbert T The thrill of being violent as an antidote to posttraumatic stress disorder in Rwandese genocide perpetrators Eur J Psychotraumatology 2011 2 6345 10.3402/ejpt.v2i0.6345 PMC3402107 22893806

[B12] Weierstall R Schalinski I Crombach A Hecker T Elbert T When combat prevents PTSD symptoms: results from a survey with former child soldiers in Northern Uganda BMJ Psychiatry 2012 12 41 10.1186/1471-244X-12-41 PMC3413590 22583755

[B13] Hecker T Hermenau K Maedl A Hinkel H Elbert T Schauer M Does Perpetrating Violence Damage Mental Health? Differences between forcibly recruited and voluntary combatants in DR Congo. Journal of Traumatic Stress J Trauma Stress 2013 26 1 142 184 10.1002/jts.21770 23319373

[B14] Theidon K Transitional subjects: the disarmament, demobilization and reintegration for former combatants in Columbia Int J Transitional Justice 2007 1 66 90 10.1093/ijtj/ijm011

[B15] Laplante LJ Theidon K Transitional justice in times of conflict: Colombia's Ley de Justicia y Paz Mich J Int Law 2006 49 107

[B16] Berdal M Disarmament and demobilization after civil wars; arms, soldiers and the termination of armed conflicts, IISS (London) Boutros-Ghali, Boutros, 1992. An agenda for peace 1996 2 New York: United Nations

[B17] Knight WA Disarmament, demobilization, and reintegration and post-conflict peace-building in Africa: an overview Afr Secur 2008 1 1 24 52 10.1080/19362200802285757

[B18] Nelson RJ Trainor BC Neural mechanisms of aggression Nat Rev Neurosci 2007 8 536 546 10.1038/nrn2174 17585306

[B19] De Posada CV Motives for the Enlistment and Demobilization of Illegal Armed Combatants in Colombia. Psychology: Peace and Conflict J Peace 2009 15 3

[B20] Schauer M Neuner F Elbert T Narrative Exposure Therapy 2011 2 Göttingen, Germany: Hogrefe & Huber

[B21] Foa EB Riggs DS Dancu CV Rothbaum BO Reliability and validity of a brief instrument for assessing post-traumatic stress disorder J Trauma Stress 1993 6 459 473 10.1002/jts.2490060405

[B22] Odenwald M Lingenfelder B Schauer M Neuner F Rockstroh B Hinkel H Screening for post-traumatic stress disorder among Somali ex- combatants: a validation study Confl Heal 2007 1 10 doi:10.1186/1752-1505-1181-1110 10.1186/1752-1505-1-10 PMC2020457 17822562

[B23] Ertl V Pfeiffer A Saile R Schauer E Elbert T Neuner F Validation of a mental health assessment in an African conflict population Int Perspect Psychol: Res, Pract, Consultation 2010 1 19 27 10.1037/a0018810 20528059

[B24] Akaike H Factor analysis and AIC Psychometrika 1987 52 3 317 332 10.1007/BF02294359

[B25] Meloy JR The psychology of wickedness: psychopathy and sadism Psychiatr Ann 1997 27 630 633

[B26] Organization of American States Eighth quaterly report of the secretary general to the permanent council on the mission to support the peace process in Colombia 2007 OEA/Ser.G; CP/doc 4176/07

[B27] Maedl A Schauer E Odenwald M Elbert T Martz E Psychological rehabilitation of ex-combatants in non-western, post-conflict settings Trauma rehabilitation after war and conflict: community and individual perspectives 2011 New York: Springer 177 214

[B28] Elbert T Rockstroh B Kolassa IT Schauer M Neuner F Baltes P, Reuter-Lorenz PF, Rösler F The Influence of Organized Violence and Terror on Brain and Mind – a Co-Constructive Perspective Lifespan development and the brain: The perspective of biocultural co-constructivism 2006 NY, US: Cambridge University Press 326 349

[B29] Elbert T Hermenau K Hecker T Weierstall R Schauer M: FORNET Endrass J, Rossegger A, Urbaniok F, Borchard B Treatment of traumatized and non-traumatized violent offenders using Narrative Exposure Therapy Interventionen bei Gewalt- und Sexualstraftätern: Risk-Management, Methoden und Konzepte der forensischen Therapie 2012 Berlin: Medizinisch Wissenschaftliche Verlagsgesellschaft

[B30] Dohrenwend BP Turner JB Turse NA Adams BG Koenen KC Marshall R The psychological risks of Vietnam for US veterans: a revisit with new data and methods Science 2006 313 979 982 10.1126/science.1128944 16917066 PMC1584215

[B31] Friedman MJ Schnurr PP McDonagh-Coyle A Posttraumatic stress disorder in the military veteran Iraqi War Clinician Guide 1994 17 2 264 277 7937358

[B32] MacNair RM Perpetration-induced traumatic stress -psychological dimensions to war and peace series 2002 Westport: Praeger

[B33] Catani C Schauer E Elbert T Missmahl I Bette JP Neuner F War trauma, child labor, and family violence: Life adversities and PTSD in a sample of school children in Kabul J Trauma Stress 2009 22 3 163 171 10.1002/jts.20415 19462436

[B34] Elbert T Schauer M Schauer E Huschka B Hirth M Neuner F Trauma-related impairment in children-A survey in Sri Lankan provinces affected by armed conflict Child Abuse Negl 2009 33 4 238 246 10.1016/j.chiabu.2008.02.008 19324413

[B35] Karunakara U Neuner F Schauer M Singh K Hill K Elbert T Burnham G Traumatic events and symptoms of post-traumatic stress disorder amongst Sudanese nationals, refugees and Ugandan nationals in the West Nile Afr Health Sci 2004 4 2 83 93 15477186 PMC2141616

[B36] Weierstall R Elbert T Endrass J, Rossegger A, Urbaniok F, Borchard B Multifaktorielle Genese und Pathologie der Aggression Interventionen bei Gewalt- und Sexualstraftätern: Risk-Management, Methoden und Konzepte der forensischen Therapie 2012 Berlin: Medizinisch Wissenschaftliche Verlagsgesellschaft

[B37] Lang PJ A bioinformational theory of emotional imagery Psychophysiology 1979 52 1048 1060 10.1111/j.1469-8986.1979.tb01511.x 515293

[B38] Foa EB Kozak MJ Emotional processing of fear: Exposure to corrective information Psychol Bull 1986 99 20 35 2871574

[B39] Colletta N Kostner M Weiderhofer I The transition from war to peace in Sub-Saharan Africa: Directions in Development 1996 Washington, DC: World Bank

[B40] Knight M Ozerdem A Guns, camps and cash: disarmament, demobilization and reinsertion of former combatants in transitions from war to peace J Peace Res 2004 41 499 516 10.1177/0022343304044479

[B41] Andersen MB Do no harm – How aid can support peace – or war 1999 London, United Kingdom: Lynne Rienner Publishers, Inc 37 53

[B42] Brzoska M Bryden A, Hänggi H Embedding DDR Programme in Security Sector Reconstruction Security Governance in Post-Conflict Peacebuilding 2005 Germany, Munster: Lit Verlag 99

[B43] Leff J The Nexus between Social Capital and Reintegration of Ex-combatants: A Case for Sierra Leone Afr J Confl Resolution 2008 8 9 38

[B44] McFate S The Link between DDR and SSR in Conflict-Affected Countries 2010 Washington, DC: United States Institute of Peace

[B45] Human Rights Watch (HRW) Herederos de los paramilitares: La nueva cara de la violencia en Colombia 2010 Nueva York: Human Rights Watch

[B46] Bettencourt BA Miller N Gender differences in aggression as a function of provocation: a meta-analysis Psychol Bull 1996 119 422 447 8668747 10.1037/0033-2909.119.3.422

[B47] Morgenstein J Consolidating disarmament: lessons from Colomibia’s reintegration program for demobilized paramilitaries U S Inst Peace, Spec Rep 2008 217

